# MUC1 Limits *Helicobacter pylori* Infection both by Steric Hindrance and by Acting as a Releasable Decoy

**DOI:** 10.1371/journal.ppat.1000617

**Published:** 2009-10-09

**Authors:** Sara K. Lindén, Yong H. Sheng, Alison L. Every, Kim M. Miles, Emma C. Skoog, Timothy H. J. Florin, Philip Sutton, Michael A. McGuckin

**Affiliations:** 1 Mucosal Diseases Program, Mater Medical Research Institute, Mater Health Services, South Brisbane, Queensland, Australia; 2 Mucosal Immunobiology and Vaccine Center, Sahlgrenska Academy, Gothenburg University, Gothenburg, Sweden; 3 Centre for Animal Biotechnology, School of Veterinary Science, University of Melbourne, Melbourne, Victoria, Australia; 4 Department of Medicine, University of Queensland, Queensland, Australia; Institut Pasteur, France

## Abstract

The bacterium *Helicobacter pylori* can cause peptic ulcer disease, gastric adenocarcinoma and MALT lymphoma. The cell-surface mucin MUC1 is a large glycoprotein which is highly expressed on the mucosal surface and limits the density of *H. pylori* in a murine infection model. We now demonstrate that by using the BabA and SabA adhesins, *H. pylori* bind MUC1 isolated from human gastric cells and MUC1 shed into gastric juice. Both *H. pylori* carrying these adhesins, and beads coated with MUC1 antibodies, induced shedding of MUC1 from MKN7 human gastric epithelial cells, and shed MUC1 was found bound to *H. pylori*. Shedding of MUC1 from non-infected cells was not mediated by the known MUC1 sheddases ADAM17 and MMP-14. However, knockdown of MMP-14 partially affected MUC1 release early in infection, whereas ADAM17 had no effect. Thus, it is likely that shedding is mediated both by proteases and by disassociation of the non-covalent interaction between the α- and β-subunits. *H. pylori* bound more readily to MUC1 depleted cells even when the bacteria lacked the BabA and SabA adhesins, showing that MUC1 inhibits attachment even when bacteria cannot bind to the mucin. Bacteria lacking both the BabA and SabA adhesins caused less apoptosis in MKN7 cells than wild-type bacteria, having a greater effect than deletion of the CagA pathogenicity gene. Deficiency of MUC1/Muc1 resulted in increased epithelial cell apoptosis, both in MKN7 cells *in vitro*, and in *H. pylori* infected mice. Thus, MUC1 protects the epithelium from non-MUC1 binding bacteria by inhibiting adhesion to the cell surface by steric hindrance, and from MUC1-binding bacteria by acting as a releasable decoy.

## Author Summary

The bacterium *Helicobacter pylori* can cause peptic ulcer disease, gastric adenocarcinoma and MALT lymphoma. *H. pylori* colonize the mucosal surface of the stomach, where adherence helps the bacteria to remain in the neutral and protected niche under the mucus layer, and helps it withstand the continuous mucus washing of the mucosal surface. Adherence is also thought to mediate much of the *H. pylori* mediated disease. The cell-surface mucin MUC1 is highly expressed on the mucosal surface and limits the density of *H. pylori* in a murine infection model. We now demonstrate that the majority of *H. pylori* strains can bind to human MUC1 and that release of MUC1 following binding limits adhesion to the cell surface. Furthermore, MUC1 protects the epithelium from non-MUC1 binding bacteria by acting as a physical barrier to adhesion to other cell surface molecules. Thus, appropriate expression and function of MUC1 is likely to limit development of disease ensuing from chronic *H. pylori* infection.

## Introduction

The bacterium *Helicobacter pylori* can cause peptic ulcer disease, gastric adenocarcinoma and MALT lymphoma [1]. *H. pylori* is estimated to cause approximately 70% of all gastric cancers, and gastric cancer is the second most common cause of cancer related deaths. *H. pylori* infection and the *H. pylori* induced pathologies, chronic atrophic gastritis and gastric cancer, are all associated with an increase in epithelial apoptosis [2],[3],[4]. One mechanism by which *H. pylori* can induce apoptosis is by the delivery of the protein CagA into epithelial cells by a type IV secretion system [5],[6]. This process subsequently activates multiple intracellular signaling cascades inducing an apoptotic response [5],[6] that has been suggested to promote gastric carcinogenesis by a compensatory increase in gastric epithelial cell proliferation [4]. Supporting this notion, there are more proliferating cells in inflamed mucosa under *H. pylori* infestation than in *H. pylori* free areas of the mucosa [7]. Furthermore, it has been shown that in response to chronic *Helicobacter felis* infection in mice, bone marrow–derived cells can home to and repopulate the gastric mucosa, replacing dead or exhausted epithelial stem cells and contribute over time to metaplasia, dysplasia, and cancer [8].

Adherence of some *H. pylori* to the mucosal surface is likely to help the bacterial population remain in the neutral and protected niche under the mucus layer, and help it withstand the continuous mucus washing of the mucosal surface. Adherence of *H. pylori* is dependent on the expression of bacterial adhesins and cognate host glycans, displayed by glycoproteins and glycosphingolipids in gastric epithelium and also by mucins in the gastric mucus layer [9],[10],[11]. Several adhesins have been implicated in *H. pylori* binding: the Blood group Antigen Binding Adhesin (BabA) binds to the fucosylated ABO/Lewis b antigen (Le^b^), and the Sialic Acid Binding Adhesin (SabA) binds to the sialyl-Lewis x (sLe^x^) and sialyl-Lewis a antigens (sLe^a^) [12],[13]. In the human stomach, the Le^b^ blood-group antigen is mainly expressed by the surface epithelium and on the MUC5AC secreted mucin [11]. Expression of sialylated Le antigens are common in infected and inflamed gastric mucosa [14],[15],[16].

Under the mucus layer, the cell-surface associated mucins are highly expressed glycoproteins on the apical surface of all mucosal epithelial cells. Because of their long filamentous nature, cell surface mucins are likely to be the first point of direct contact between host tissue and organisms that penetrate the secreted mucus layer. MUC1 is the most highly expressed cell surface mucin in the stomach [17]. Most mucins exhibit considerable genetic polymorphism due to variability in their numbers of tandem repeat peptides, which results in proteins of widely divergent lengths. Several studies have linked *MUC1* polymorphisms with susceptibility to *H. pylori*-induced disease, such as gastritis and gastric cancer [18],[19], suggesting a direct effect of *MUC1* polymorphisms on the development of *Helicobacter*-associated pathology. We have shown that mice deficient in Muc1 are more susceptible to infection by both *H. pylori* [20] and *Campylobacter jejuni* [21]. However, the mechanism by which this mucin limits bacterial pathogenesis has not been elucidated. In this study, we have characterized mechanisms by which MUC1 limits *H. pylori* colonization and decreases ensuing mucosal pathology.

## Results

### MUC1 is shed from the epithelial cell surface into the gastric juice and carries the Le^b^, sLe^x^ and sLe^a^ carbohydrate structures

**Figure 1:**
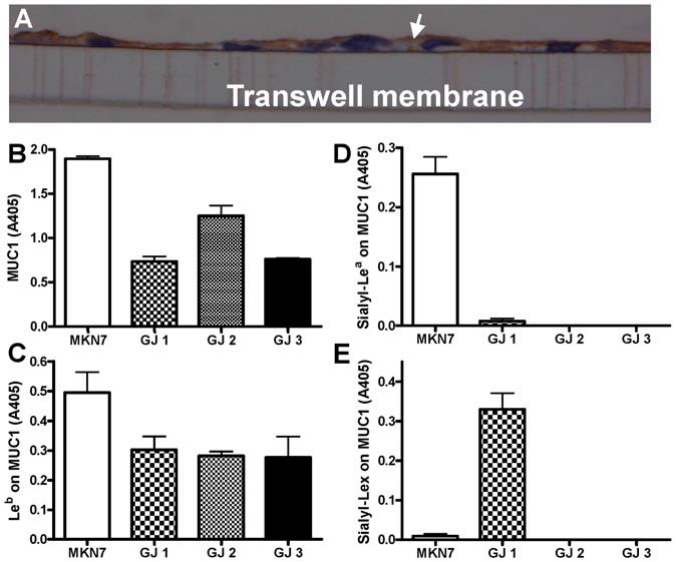
Expression and glycosylation of cell associated and shed MUC1. (A) Demonstration of MUC1 expression on the apical surface (arrow) of MKN7 gastric cancer cells cultured on a transwell membrane for 3 days post confluency by staining with an anti-MUC1 cytoplasmic domain antibody (CT2). (B–E) Cell associated and shed MUC1 was captured from the high molecular weight fraction of a cellular lysate from MKN7 cells and from three human gastric juice samples (GJ), using a microtitre plate coated with an antibody against the extracellular domain of MUC1 (BC2). Bound MUC1 was detected with another antibody against the extracellular domain of MUC1 (BC3) (B), and the presence of the Le^b^ (C), sialyl-Le^a^ (D) and sialyl-Le^x^ (E) carbohydrate structures on MUC1 were analyzed using antibodies against these structures. Statistics: mean±SEM after subtraction of the mean value of the isotype control coated wells for that sample.

The human gastric epithelial cell line MKN7 forms a contiguous polarized monolayer (epithelial resistance 250 ohm/cm^2^) and expresses MUC1 on the apical membrane surface as occurs in normal gastric epithelium (Figure 1A). Using double determinant ELISA we measured MUC1 in MKN7 cells and human gastric juice (Figure 1B), demonstrating that MUC1 is shed from the gastric cell surface *in vivo*. MUC1 from the MKN7 cells carried the Le^b^ and sLe^a^ oligosaccharide structures (Figure 1C, D) but not sLe^x^ (Figure 1E) demonstrating that MKN7 MUC1 possesses ligands for both the SabA and BabA *H. pylori* adhesins. The glycosylation of MUC1 in gastric juice varied between individuals. Shed MUC1 from all three individuals tested carried Le^b^ (Figure 1C), MUC1 from one individual carried sLe^x^ (Figure 1E) but none of the three gastric juice samples we investigated carried sLe^a^ on MUC1 (Figure 1D). This is consistent with interindividual differences in glycosylation demonstrated for secreted gastric mucins such as MUC5AC [11].

### *H. pylori* binds to MUC1 carbohydrate structures and binding is dependent on *H. pylori* adhesins

**Figure 2:**
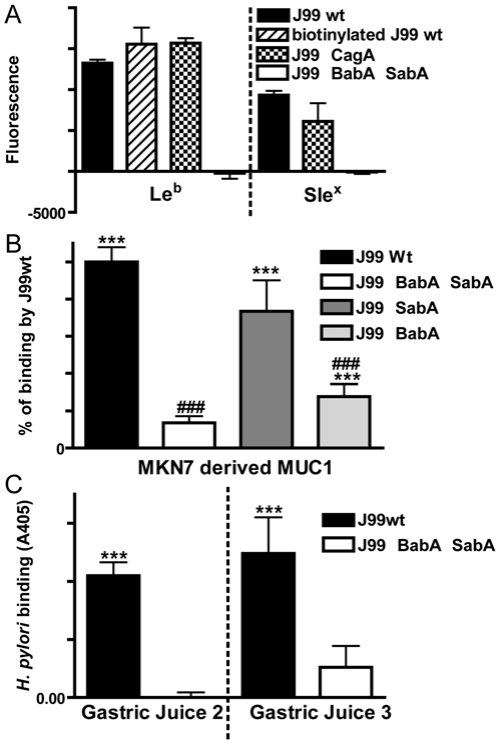
*H. pylori* binds to synthetic glycoconjugates, cell associated MUC1 and shed MUC1 via the BabA and SabA adhesins. Adhesion of *H. pylori* J99 wild type (wt), biotinylated J99wt, a ΔCagA isogenic mutant and a ΔBabASabA isogenic double mutant to FITC labelled Le^b^ or sialyl-Le^x^ conjugated to human serum albumin (A). Cell associated and shed MUC1 were isolated using a microtitre plate coated with the BC2 antibody against the extracellular domain of MUC1 (or isotype control) to capture MUC1 from the high molecular weight fraction of a cellular lysate from the MKN7 gastric cancer cell line (B) and from two human gastric juice samples (C), respectively. Biotinylated J99wt (B, C) or the isogenic adhesion mutants J99ΔBabA (B), J99ΔSabA (B) or a J99ΔBabAΔSabA double mutant (B, C), were added to the wells where MUC1 had been captured by the anti-MUC1 antibody (or isotype control) and binding was detected with streptavidin-HRP. Statistics: mean±SEM after subtraction of the mean value of bacteria-only (A) or isotype control (B-C) coated wells for that sample; ANOVA with Bonferroni post-hoc test (B) or Students t-test (C), N = 6; vs isotype control (B,C ** p<0.01, *** p<0.001;); vs J99wt (B, ### p<0.001).

*H. pylori* strains differ in their expression of the BabA and SabA adhesins and consequently in their ability to bind to the Le^b^, sLe^a^ and sLe^x^ carbohydrate structures expressed on gastric mucins [11],[13]. The J99 wild type strain possesses both adhesins, and bound to the adhesion ligands Le^b^ and sLe^x^ (Figure 2A). J99 bound to MUC1 isolated from the human gastric epithelial cell line MKN7 (Figure 2B) as well as to MUC1 shed into human gastric juice in both samples that were tested (Figure 2C), as analyzed using a double determinant ELISA. This demonstrates that *H. pylori* can bind to both cell associated and shed MUC1. In contrast, when an isogenic J99 double mutant lacking both BabA and SabA was used, no specific attachment of the bacteria to MUC1 was detected (Figure 2B and C). A single J99 SabA deletion mutant resulted in a small non-significant decrease in MUC1 binding and a single J99 BabA deletion mutant showed intermediate MUC1 binding (Figure 2B). This demonstrates that binding to MUC1 occurred via MUC1 oligosaccharides bound by both the BabA and SabA adhesins. Since the healthy stomach normally contains a very low amount of sialylated structures, further binding studies were performed with Le^b^ positive and sLe^x^ negative MUC1 obtained from a healthy gastric surgical resection specimen. The BabA positive *H. pylori* strains P466 and CCUG17875/Leb also bound to MUC1, whereas the BabA negative strains 75ΔBabA1/A2, CCUG17874, 26695, P1, P1-140 and M019 did not (Figure S1). Thus, non-MUC1 binding *H. pylori* strains also exist. However, 80% of *H. pylori* strains are BabA positive [12], and the majority of *H. pylori* strains thus have the ability to bind to MUC1.

### MUC1 acts as a releasable decoy for MUC1-binding bacteria-sized beads

**Figure 3:**
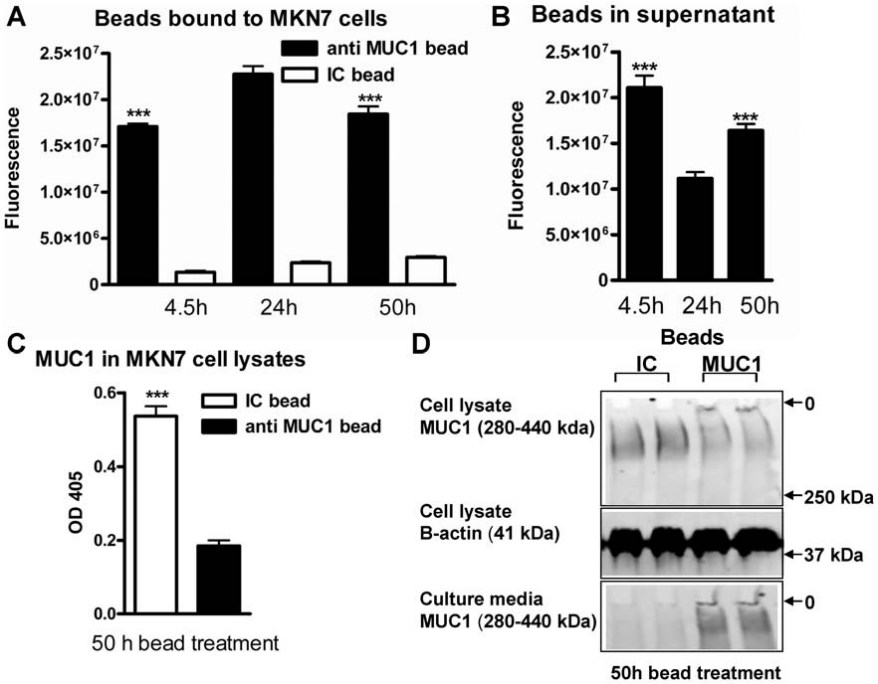
MUC1 is released from the epithelial surface upon adherence of MUC1-binding beads. 10^5^ fluorescent beads coated with the BC2 antibody against the extracellular domain of MUC1 or an isotype control (IC) were added to 96 well plates with confluent MKN7 epithelial cells and cultured for up to 50 h. Binding was assessed after 4.5, 24 and 50 h by determining fluorescence of washed monolayers (A) and culture supernatants (B). Statistics: Mean ± SEM, n = 9; ***p<0.001 vs 24 h. (C) The amount of MUC1 α-subunit (extracellular domain) in cells treated with anti-MUC1 coated beads compared to isotype control (IC) coated beads was detected by double determinant ELISA using the BC2 (capture) and BC3 (detection) antibodies (***p<0.001). Western blotting (D) was used to detect the MUC1 extracellular domain (using the BC2 antibody) in cell lysates from cells treated with anti-MUC1 coated beads compared to IC for 50 h (top panel, MUC1 has an apparent mobility in the range of 280–440 kDa due to its polydisperse glycosylation). β-actin as a loading/cell density control in the same gel is shown in the middle panel. Concentrated culture supernatants from the same cell monolayers run on a separate SDS-PAGE/Western blot are shown in the lower panel. Origins of the gels are indicated (O).

The demonstration that *H. pylori* binds to MUC1 and that MUC1 can be shed from the epithelial surface indicated that MUC1 could act as a releasable decoy upon bacterial adherence. To determine whether MUC1 release can be triggered simply by particulate binding, in the absence of microbial molecular signalling, we used 1 µm beads coated with either an antibody against the extracellular domain of MUC1 or with an isotype control antibody. Immunohistochemistry of cell layers incubated with the beads demonstrated that the MUC1-antibody-coated beads attached to the cell surface but that they were not endocytosed (data not shown). Adherence of beads coated with the anti-MUC1 antibody to confluent MKN7 epithelial cells was significantly higher at 24 h than at 4.5 h and then decreased (Figure 3A). This pattern is consistent with a loss of beads from the cell surface once the MUC1 binding sites on the beads are filled and/or the MUC1 on the epithelial cell surface is depleted. The amount of beads in the conditioned culture supernatant followed the opposite pattern, demonstrating that the decrease of beads attached to the cells is due to release into the supernatant rather than bleaching of the fluorescence (Figure 3B). Furthermore, the amount of cellular MUC1 α-subunit (extracellular domain) was decreased in cells treated with anti-MUC1 coated beads compared to isotype control coated beads, as demonstrated by capture ELISA (Figure 3C). This was not due to the adherent beads sterically hindering antibody access to MUC1 on the cell surface, as the same decrease was shown by Western blotting of cell lysates (Figure 3D). MUC1 was also detected in the bead-containing conditioned media from cells treated with anti-MUC1 coated beads, but not with isotype control coated beads (Figure 3D). These results demonstrate that when inert bacterial sized particles bind to MUC1, the MUC1 extracellular domain is released from the epithelial cell surface.

### Infection results in progressive depletion of cellular MUC1 and binding of MUC1 to *H. pylori*

**Figure 4:**
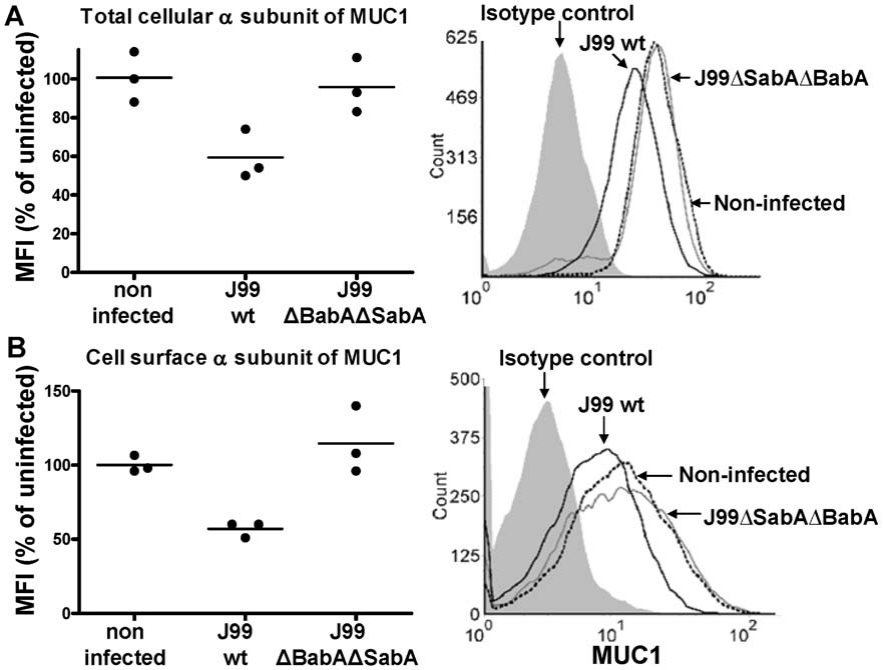
Cell surface MUC1 is depleted during *H. pylori* infection *in vitro*. The concentration of cellular MUC1 from non-infected MKN7 cells and cells co-cultured with *H. pylori* J99 wild type or J99ΔBabAΔSabA (MOI 1∶1) for 44 h was determined by flow cytometry (with the BC2 antibody) in order to measure the amount of MUC1 per viable cell (the analysis was gated to exclude 7AAD positive dead cells) and distinguish between the total amount of α subunit (the extracellular domain of MUC1) determined in permeabilized cells (A) and the amount of α subunit on the cell surface (B). Representative histograms from the flow cytometry analysis are shown on the right. Median fluorescence intensity (MFI) was determined after subtraction of the MFI using a negative control antibody. Results from 3 independent replicate cultures are shown, expressed as a percentage of the mean MFI of cells from non-infected cultures.

Co-cultures of confluent MKN7 cells and *H. pylori* were established in microaerobic conditions at 1∶1–1∶20 (mammalian cell∶bacteria:) as a model of the interaction between the gastric epithelium and *H. pylori* that penetrate gastric mucus. Under microaerobic conditions the mammalian cells are not stressed and the *H. pylori* survive, proliferate and show a closer physiology to that seen *in vivo* than the typically used aerobic co-cultures [22]. The use of confluent cultures is important in modelling the gastric epithelium to avoid *H. pylori* binding to the basolateral surface of cells, as occurs with the typically used non-confluent AGS culture system [23]. MUC1 is polarised to the apical membrane in these MKN7 cultures (Figure 1A). After 44 h of co-culture we used flow cytometry to measure the amount of cell surface and total MUC1 within individual viable MKN7 cells. Co-culture with the *H. pylori* strain J99wt depleted MKN7 cells of approximately 40% of both the total pool of the extracellular domain of MUC1 (Figure 4A) and of the cell surface located extracellular domain of MUC1 (Figure 4B). To explore whether *H. pylori* SabA or BabA adhesin mediated binding to MUC1 was required for depletion of MUC1 we used the J99ΔBabAΔSabA mutant as the single mutants were able to bind MUC1 (Figure 2B). Co-culture with the double adhesin mutant did not deplete cellular or cell surface MUC1. These results are consistent with decreased MUC1 production, increased degradation in the cell or release of MUC1 following binding of the live MUC1-binding bacteria in a similar manner to that shown with the inert 1 µm beads.

**Figure 5:**
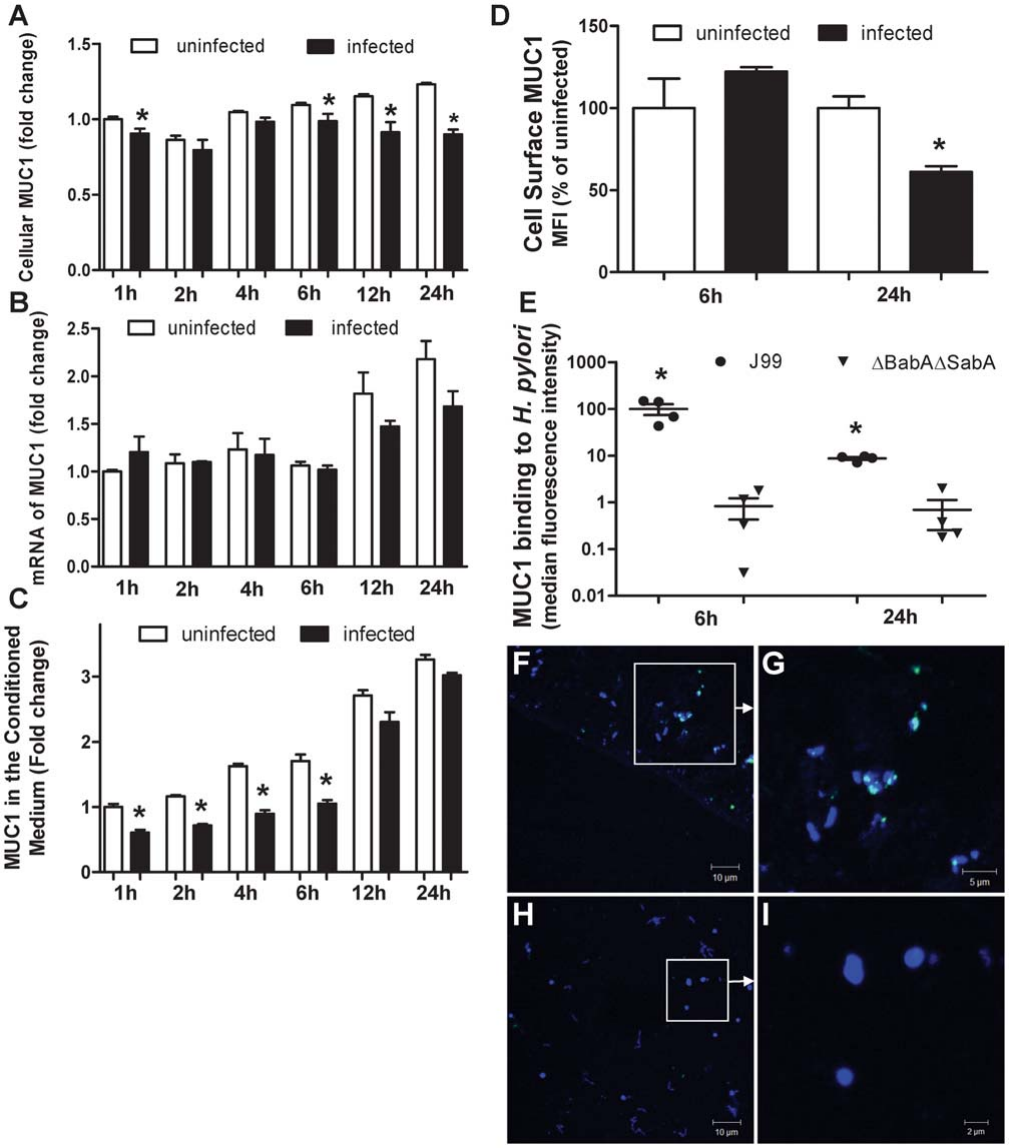
MUC1 is shed from the cell surface and binds *H. pylori* following co-culture *in vitro*. MKN7 cells were uninfected or co-cultured with *H. pylori* J99 wild type for 1, 2, 4, 6, 12 and 24 h (MOI 10∶1) and total cellular MUC1 determined by BC2/BC3 double determinant ELISA as in Figure 3C (A), MUC1 mRNA concentrations relative to GAPDH were determined by qPCR (B), and concentrations of free MUC1 in conditioned medium determined by ELISA (C). In a further experiment after co-culture of MKN7 cells with *H. pylori* J99 wild type for 6 and 24 h cell surface MUC1 was assessed in MKN7 cells by flow cytometry as in Figure 4B (D). In a similar experiment both the *J99* wild type (MOI 10∶1) and the J99ΔBabAΔSabA (20∶1) were co-cultured with MKN7 cells for 6 and 24 h and the presence of MUC1 on bacteria recovered from the conditioned media was determined by staining with the BC2 antibody or an isotype control followed by flow cytometry (E) and confocal microscopy (F–I). For flow cytometry the MFI of the isotype control was subtracted from the MFI of BC2-stained bacteria for each replicate culture. For confocal microscopy, *H. pylori* J99 wild type (F, G) or J99ΔBabAΔSabA (H, I) were stained with BC2 (FITC-conjugated secondary antibody, green), DNA (DAPI, blue); scale bars are shown (single scans and additional controls are shown in Figure S2). Scale bars are shown. Statistics: mean±SEM from 4 independent replicate co-cultures; Non-parametric test (Mann Whitney); vs non-infected at same time point (A, C, D) vs J99ΔBabASabA (E), * p<0.05.

Further experiments over 1–24 h showed that the decrease in cellular MUC1 began early in co-culture (Figure 5A; cellular MUC1 was lower in infected cells at 1, 6, 12 and 24 h). However, MUC1 mRNA levels increased progressively in culture but were not significantly affected by the presence of *H. pylori* (Figure 5B). The rapid decrease in MUC1 protein with sustained levels of *MUC1* mRNA, suggest that loss of protein rather than decreased MUC1 production is the likely explanation for MUC1 depletion from the cell. Despite the unchanged mRNA levels and the decreasing cellular MUC1, the amount of free MUC1 in the conditioned medium was significantly lower (by about 40%) in infected cultures at 1, 2, 4 and 6 h (Figure 5C). Decreased MUC1 in the culture medium in infected cultures suggests that substantial amounts of MUC1 was either bound to the bacteria (which were removed by centrifugation prior to ELISA) and/or was degraded. In a further experiment, total and cell surface MUC1 was assessed by flow cytometry after 6 and 24 h of co-culture. After 6 h of co-culture *H. pylori* strain J99 wt did not affect the level of MUC1 on the cell surface, however, it led to depletion of 44% of cell surface MUC1 after 24 h infection compared to uninfected MKN7 cells (Figure 5D). Bacteria recovered from the culture medium were stained for the presence of MUC1 using an antibody reactive with the extracellular domain. MUC1 was detected bound to *H. pylori* strain J99wt but not to the J99ΔBabAΔSabA mutant using both flow cytometry (Figure 5E) and confocal microscopy (Figure 5F–I, Figure S2). Binding to *H. pylori* strain J99wt was greatest at 6h (Figure 5E) suggesting the bacteria may be capable of degrading or shedding bound MUC1. Confocal microscopy showed that MUC1 binding to bacteria was often focally intense rather than evenly distributed on the bacterial surface (Figures 5F,G). Taken together these data are consistent with progressive shedding of MUC1 from the cell surface and binding of shed MUC1 to bacteria dependent on the BabA and SabA adhesins.

### MUC1 shedding is partially dependent on MMP-14

**Figure 6:**
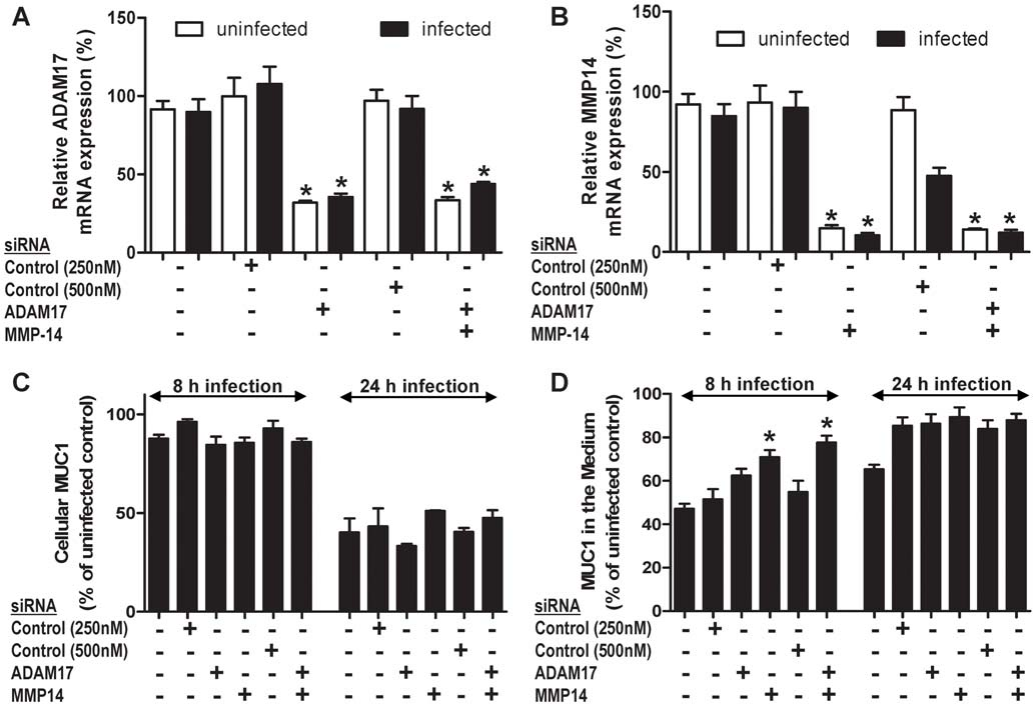
Shedding of MUC1 is partially dependent on the MMP-14 protease. MKN7 cells were transfected with control (scrambled), ADAM17, MMP-14 or ADAM17 plus MMP-14 siRNA. Following 48 h of the transfection, MKN7 cells were infected with *H. pylori* J99 wild type or cultured uninfected for a further 8 and 24 h. Expression of ADAM17 (A) and MMP-14 (B) mRNA relative to GAPDH was determined by qPCR in uninfected or *H. pylori* J99 wild type infected cultures (8 h infection, MOI 10∶1). After 8 and 24 h of infection concentrations of cellular MUC1 (C) and free MUC1 in conditioned media (D) were determined by BC2/BC3 double determinant ELISA as in Figure 3C. MUC1 concentrations after infection are presented as a percentage of the mean of the uninfected control for each transfection. Statistics: mean ± SEM from 4 independent replicate co-cultures; Nonparametric test (Mann Whitney); vs scrambled si RNA negative control (* p<0.05).

**Figure 7:**
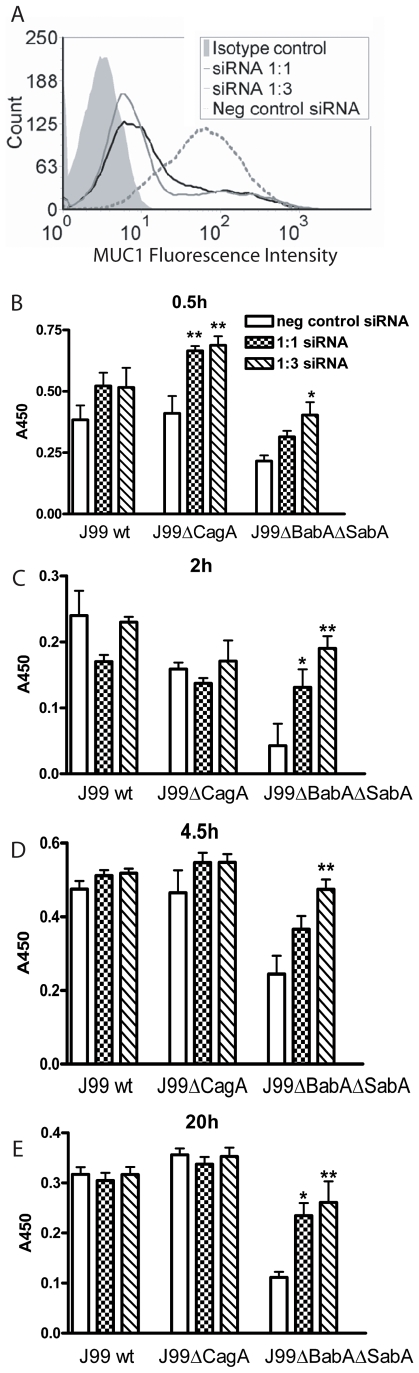
Increased adherence of *H. pylori* to gastric epithelial cells with reduced Muc1 expression. (A) Transfection of siRNAs into MKN7 epithelial cells reduced MUC1 expression on the cell surface by 80% with the 1∶1 sequence and 85% with the 1∶3 sequence compared to scrambled siRNA as determined by flow cytometry with an extracellular domain antibody BC2. (B–E) *H. pylori* J99wt, the CagA deletion mutant J99ΔCagA or adhesion mutant J99ΔBabAΔSabA were co-cultured at a concentration of 8×10^5^ CFU *H. pylori*/well (MOI 16∶1) in a 96 well plate for 0.5 (B), 2 (C), 4.5 (D) and 20 h (E). Adhesion of *H. pylori* to cultured cells was determined by ELISA. Data are presented after subtraction of the mean value of the OD for gastric cells without *H. pylori*. Statistics: Mean±SEM (N = 6); ANOVA, post hoc: Tukey's test; scrambled siRNA vs MUC1 specific siRNA * p<0.05, ** p<0.01.

**Figure 8:**
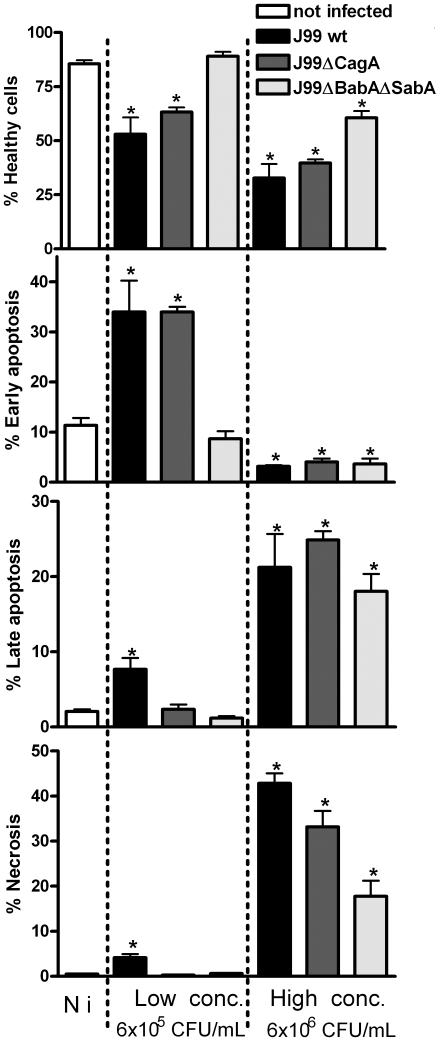
*H. pylori* induced apoptosis and necrosis is affected by both adhesion and CagA. MKN7 cells (Not infected; N i, left column) co-cultured with either 6×10^5^ CFU/mL (MOI 1∶1, middle column) or 6×10^6^ CFU/mL (MOI 10∶1, right column) *H. pylori* (in 500 µl) for 20 h. The graphs show the percentage of healthy cells, cells in early apoptosis (Annexin-V^+^, 7-AAD^−^), late apoptosis (Annexin-V^+^, 7-AAD^+^ cells) and necrosis (Annexin-V^−^, 7-AAD^+^ cells) analyzed by flow cytometry. Statistics: Mean ± SD from 3 independent replicate co-cultures; Mann Whitney U test, vs non-infected * p<0.05.

**Figure 9:**
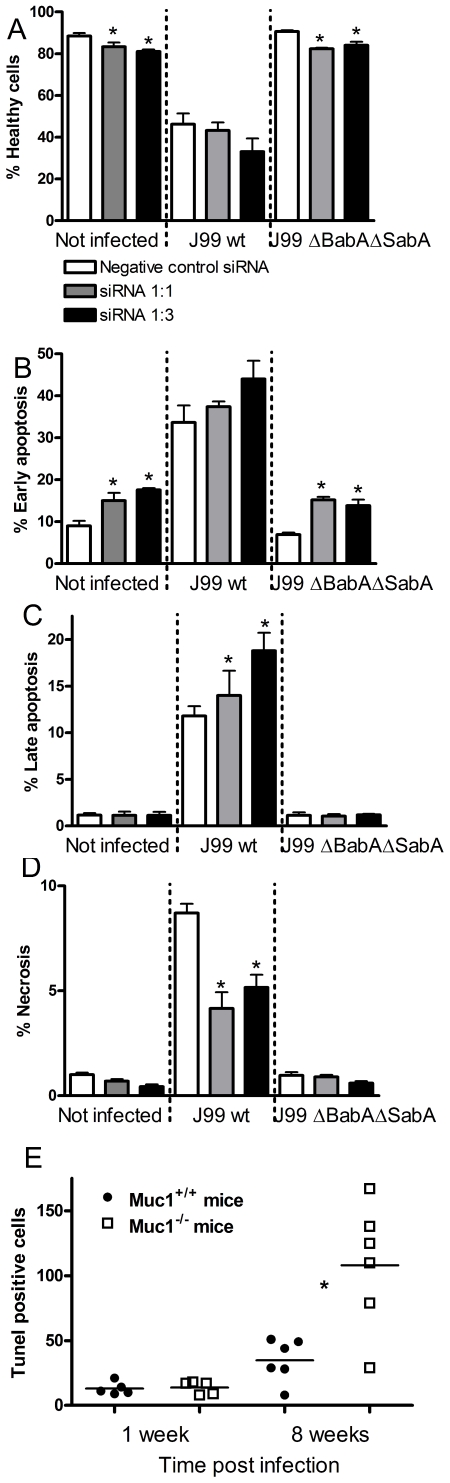
Gastric epithelial cell apoptosis and necrosis is affected by MUC1/Muc1. (A–D) MKN7 transfected with MUC1 siRNAs 1∶1, 1∶3 (97 and 93% knockdown compared to scrambled siRNA) and scrambled siRNA were co-cultured with 3×10^5^ *H. pylori*/well (500 µL, MOI 1∶1) for 20 h (A–D). The graphs show (A) the percentage of healthy cells, (B) cells in early apoptosis (Annexin-V^+^, 7-AAD^−^), (C) late apoptosis (Annexin-V^+^, 7-AAD^+^ cells), and (D) necrosis (Annexin-V^−^, 7-AAD^+^ cells), analyzed by flow cytometry. Data were collected for three independent replicate co-cultures and are presented as mean±SD. (E) Six wild-type (*Muc1^+/+^*) and *Muc1^−/−^* mice were infected with *H. pylori*-SS1 as previously described [20]. The number of TUNEL positive cells per 10 random fields of view in the gastric mucosa was counted at 20× magnification. Statistics: bar = mean, Mann Whitney U test, * p<0.05. Photomicrographs of the TUNEL stained sections are shown in Figure S3.

Release of MUC1 can occur either by (a) breakdown of the non-covalent association of the α- and β-subunits, possibly in response to shear stress or conformational changes, and/or (b) action of extracellular proteases/sheddases. The proteases ADAM17 and MMP-14 have previously been shown to act as MUC1 sheddases in endometrial cells [24],[25]. As both of these proteases are expressed by MKN7 cells we repeated co-culture experiments with and without knockdown of expression of either and both ADAM17 and MMP-14 using siRNA. Successful knockdown of mRNA expression of the proteases was achieved over a time period from 8 h (Figures 6A,B) to 24 h (data not shown) after infection. Cellular MUC1 decreased with infection at 24 h and free MUC1 in the culture medium also decreased at 8 h which is consistent with other experiments (presented as the proportion of uninfected controls for all siRNA conditions in Figure 6C and D, respectively). There were no significant alterations in the amount of cellular MUC1 in either uninfected or infected MKN7 cells following knockdown of ADAM 17 or MMP-14. However, there were significant increases in the amount of shed MUC1 following knockdown of MMP-14 at 8 but not 24 h of infection, although no changes were seen following knockdown of ADAM17 (Figure 6D). These data indicate that MMP-14 is partially involved in the MUC1 shedding process in MKN7 cells.

### Increased adherence of *H. pylori* to gastric epithelial cells lacking MUC1

Transfection of MUC1 siRNAs into MKN7 epithelial cells reduced cell surface MUC1 expression by 80% with the 1∶1 siRNA and by 85% with the 1∶3 siRNA, compared to scrambled siRNA (Figure 7A). *H. pylori* J99wt, J99ΔCagA or J99ΔBabAΔSabA (8×10^5^ CFU *H. pylori*/well) were co-cultured with confluent transfected MKN7 monolayers for 0.5, 2, 4.5 and 20 h. While the J99wt and J99ΔCagA strains bound MKN7 cells to a similar level, binding of the J99ΔBabAΔSabA adhesin mutant was reduced at all time points (as determined by staining the co-cultures with an anti-*H. pylori* antibody, Figure 7B–E; p<0.001). After 30 min of co-culture, binding of J99 (both with and without the SabA and BabA adhesins) to MKN7 cells was higher when MUC1 was depleted by siRNA (Figure 7B). By 2 h, and through to 20 h, siRNA knockdown of MUC1 had no significant effect on adhesion to the epithelial cells of the J99wt and J99ΔCagA strains that bind MUC1 via the SabA and BabA adhesins (see Figure 2). In contrast, MUC1 protection against adhesion of the J99ΔBabAΔSabA strain (which did not bind MUC1, see Figure 2) to the epithelial cells was maintained to at least 20 h. When considered together with the changes in MUC1 expression shown in Figures 4 and 5, these results indicate that: (a) while binding via the BabA and/or SabA adhesins increases *H. pylori* adhesion to gastric epithelial cells, these bacteria can bind epithelial cells via mechanisms other than these adhesins; (b) during early infection, MUC1 inhibits *H. pylori* binding to epithelial cells occurring via both adhesin-dependent and -independent mechanisms; (c) as infection progresses MUC1 is depleted from the epithelial cell surface of cells infected with *H. pylori* strains carrying the BabA and SabA adhesins and is no longer effective in inhibiting attachment; and (d) in cells that are infected with *H. pylori* strains that cannot bind to MUC1, MUC1 continues to be protective against adhesion to the epithelial cells, most likely by steric hindrance of bacterial interactions with non-MUC1 receptors expressed on the epithelial cell surface.

### Apoptosis and necrosis is affected by *H. pylori* adhesion, CagA and MUC1

To analyze cell death we used flow cytometry in combination with annexinV/7-AAD staining which can distinguish healthy cells, cells in early apoptosis, late apoptosis and necrosis/very late apoptosis. The extent of *H. pylori* induced apoptosis in MKN7 cells was dependent on the concentration of bacteria in the co-cultures. In MKN7 cells co-cultured for 20 h with 6×10^5^ CFU/mL *H. pylori* (MOI 1∶1), J99wt induced 35% early apoptosis, 8% late apoptosis and 5% necrosis as determined by annexinV/7AAD staining. J99ΔCagA induced similar levels of early apoptosis, but no late apoptosis or necrosis suggesting a delayed induction of apoptosis, while J99ΔBabAΔSabA had no affect on the viability of MKN7 cells (Figure 8A–D, left columns). At a 10-fold higher concentration (6×10^6^ *H. pylori* CFU/mL, MOI 10∶1), all the isogenic strains induced more apoptosis and necrosis compared with the lower concentration of *H. pylori*, albeit in a similar pattern; J99wt induced more cell death than J99ΔCagA, and J99ΔCagA induced more cell death than J99ΔBabAΔSabA (Figure 8A–D, right panel). Thus, both BabA/SabA mediated adhesion to the epithelial cell and CagA contribute to epithelial cell death, but to get the maximal effect of CagA, *H. pylori* needs to bind to the epithelial cell via the BabA and SabA adhesins.

In an experiment to evaluate the importance of MUC1 in *H. pylori* induced apoptosis, transfection of siRNAs into MKN7 cells reduced MUC1 levels by 93% and 97% with the 1∶1 and 1∶3 sequences respectively, compared to scrambled siRNA. Cultures with depleted MUC1 had fewer healthy cells and more apoptotic cells, irrespective of whether or not they were infected (Figure 9A–D). However, the proportion of necrotic cells was slightly lower in the cells with reduced MUC1.

Finally, to ascertain whether MUC1/Muc1 can also influence *H. pylori* induced epithelial apoptosis *in vivo* we analysed apoptosis in wild-type (*Muc1^+/+^*) and *Muc1^−/−^* mice infected with *H. pylori*-SS1 as previously described [20]. While no difference was observed 1 week post-infection, *Muc1^−/−^* mice had a 3-fold increase in the mean proportion of TUNEL-positive apoptotic cells in their gastric mucosa after 8 weeks of *H. pylori* infection, compared to *Muc1^+/+^* mice (Figures 9E and S3). Thus, MUC1/Muc1 protects against apoptosis both *in vitro* and in a murine *H. pylori* infection model.

## Discussion

Previously we have shown that mice deficient in Muc1 are more susceptible to infection by *H. pylori* [20] both with regard to the level of colonisation and the degree of pathology that develops. We have now extended these observations by examining the mechanism by which this mucin can limit infection by *H. pylori*. Here we demonstrate that MUC1 protects the epithelial cell from both bacterial adhesion and apoptosis. When the pathogen does not bind to MUC1, the 200–500 nm long extracellular domain of the mucin appears capable of physically distancing the bacteria from the host cell surface, thus sterically inhibiting adhesion to other potential cell surface ligands. When the pathogen does bind to MUC1, the extracellular domain of the mucin is released from the epithelial surface, thereby acting as a releasable decoy and preventing prolonged adherence. In the process *H. pylori* are coated with MUC1 via, as we demonstrate here, interactions between the mucin and the BabA and SabA adhesins. We propose that this would further prevent anchorage to the mucosal surface by blocking these key adhesins. Limiting attachment of the bacteria to the epithelial surface would also be expected to reduce pathogenicity, by restricting the functional activity of secretion systems such as that encoded by the Cag pathogenicity island, which delivers proinflammatory mediators into epithelial cells [26].

An isogenic CagA deletion mutant induced less cell death in the polarized MKN7 cultures we established in microaerophilic conditions, consistent with previous studies in other cell lines [27]. However, in our experiments which are the first to test the influence of SabA and BabA adhesins on apoptosis empirically, ablation of both adhesins had a substantially greater impact on *H. pylori* induced apoptosis than loss of CagA. Thus, both adhesion to the epithelial cell and CagA affect viability, but to get the maximal effect of CagA, *H. pylori* needs to bind to the epithelial cell via the lectin adhesins. Interestingly, at very high non-physiological bacterial infection densities, similar to those used by most researchers, these nuances were lost. Infection with BabA positive *H. pylori* strains has been associated with higher lymphocytic infiltration, increased epithelial proliferation, and the presence of glandular atrophy and intestinal metaplasia in human antral biopsies [28]. Similarly, severe neutrophil infiltration and atrophy (important indicators of more severe pathology) are associated with the expression of functional SabA [29]. Here, we showed that *H. pylori* induces apoptosis and necrosis in gastric epithelial cells in a dose dependent manner, and that an isogenic mutant strain lacking the BabA and SabA adhesins had diminished ability to induce cell death. This indicates that these adhesins are major determinants of cell surface binding and that it is the amount of adherent *H. pylori* that determines the impact on epithelial cell viability.

To inhibit bacterial access to the epithelial cells, the mucosal surfaces are covered with a mucus layer primarily composed of secreted mucins. This mucus layer is continuously secreted and transports away trapped material. Both salivary and gastric mucins have a high and specific binding capacity for *H. pylori* [9],[10],[11], and it is likely that this mucus layer keeps the majority of *H. pylori* away from the epithelial cell surface. For example, in the human-like rhesus monkey model [30], monkeys secreting mucins with less *H. pylori* binding capacity develop higher *H. pylori* density infections and gastritis [14]. Similarly, humans with primary Sjogren's syndrome, who produce less mucins, have more *H. pylori*-associated pathology [31], suggesting that the ability of secreted mucins to bind to *H. pylori* protects the gastric epithelium. In cultured MKN7 cells we observed a progressive depletion of MUC1 as it was shed off the cell surface. In human patients with chronic gastritis, depletion of the MUC1 extracellular domain α-subunit, but not the transmembrane β-subunit, has been reported [32]. Thus, it is likely that during chronic infection rapid shedding of the extracellular domain of MUC1 occurs *in vivo*, producing a similar result to that we observed *in vitro*.

We have shown that *H. pylori* adherence to live gastric epithelial cells is increased when the bacterial strain carries the BabA and SabA adhesins. However, adherence to live epithelial cells occurs even when these adhesins are absent, albeit to a reduced level. In contrast, binding to MUC1, as we have shown previously for other mucins [10],[11], is dependent on the presence of *H. pylori* adhesins. During the initial contact with gastric cells, MUC1 inhibits adhesion of both MUC1-binding and non MUC1-binding *H. pylori* to epithelial cells. *H. pylori* bind to MUC1 isolated from epithelial cells as well as to MUC1 shed into the gastric juice of human patients. In this study we have shown that, *in vitro*, *H. pylori* carrying these adhesins caused the gastric epithelial cells to shed MUC1 which bound to the bacteria. Inert bacterial sized beads coated with MUC1 antibodies also caused shedding of MUC1, demonstrating that MUC1 can act as a releasable decoy following engagement by particulate ligands in a Toll like receptor independent manner. Our demonstration of progressive depletion of cellular MUC1 is consistent with a previous study where MUC1 expression decreased in Kato III cells after 4 h of co-culture with *H. pylori* (with unknown adhesion properties) [33]. Although the study with Kato III cells showed recovery of MUC1 after 24 h of co-culture, the recovery of MUC1 may be because this study added 1000-fold higher concentrations of *H. pylori* than used in our study, and their *H. pylori* died during the experiment according to the authors possibly due to the use of aerobic culture conditions [22],[33],[34].

We have demonstrated that MMP-14 but not ADAM17 is the relevant protease involved in the endogenous shedding of MUC1 from MKN7 cells. This is consistent with previous reports that found that MUC1 shedding was sensitive to MMP-14 depletion in endometrial cells [24]. However, MMP-14 only partially affected MUC1 shedding during infection and had no influence on MUC1 shedding in non-infected cells, indicating that other factors are involved in MUC1 release. Although cleavage by another unknown MUC1 sheddase cannot be definitively excluded, the most likely scenario is disassociation of the non-covalent interaction between the transmembrane and extracellular domains at the SEA module, a site of cleavage during synthesis found in most cell surface mucins [35],[36]. Disassociation could occur due to conformational changes in MUC1 following binding or due to shear forces following binding to the highly motile bacteria. Additionally, secreted isoforms of *MUC1* expressed via alternative splicing [37], could potentially be shed without prior *H. pylori* binding and contribute to the MUC1 pool in the mucus and gastric juice. The pattern of focal binding of MUC1 to bacteria that we observed by confocal microscopy is consistent with binding of MUC1 to regions of the bacteria coming in contact with the cell surface, followed by MUC1 shedding and bacterial detachment. However, this pattern could also arise if the SabA and BabA adhesins are focally expressed or if they are aggregated on the bacterial surface following ligation by MUC1.

MUC1 confers resistance to apoptosis induced by genotoxic drugs, the *Campylobacter jejuni* cytolethal distending toxin and oxidative stress *in vitro* [21],[38]. We found that *Muc1^−/−^* mice infected with *H. pylori* strain SS1 had substantially more apoptotic cells in their gastric mucosa than *Muc1^+/+^* mice. *In vitro*, cultures with MUC1 knockdown had a higher proportion of apoptotic cells, both when infected and not infected, indicating that epithelial cell apoptosis is down regulated by MUC1 as a general function, not only as a response to stressors. Paradoxically, however, necrosis was lower in cells with reduced MUC1. A similar increase of damage induced apoptosis and delay of secondary necrosis has previously been described for mono(ADP-ribosyl)transferases, which control signal transduction pathways in response to cell damage during cell repair and apoptosis [39]. MUC1 increases ß-catenin levels in the cytoplasm and nuclei of carcinoma cells by blocking its degradation, resulting in an increase in cell proliferation [40],[41]. Similarly, NF-κB regulates genes that control cell proliferation and cell survival, and NF-κB is constitutively active in some cancers. MUC1 interacts directly with the IκB kinase complex, resulting in degradation of the NF-κB inhibitor IκBα [42]. Thus, high expression of MUC1, as occurs in the stomach and is commonly found in human cancers, confers increased proliferation and resistance to apoptosis both via ß-catenin and the NF-κB pathway. NF-κB is also the master regulator of inflammatory responses, including responses to *H. pylori*. In addition to a possible effect on NF-κB, it is possible that MUC1 modulates responses to PAMPs mediated by TLRs. In respiratory cells, MUC1 binding of bacterial flagellin (TLR5 ligand) appears to repress TLR5-mediated activation of inflammation [43],[44]. Thus, in addition to limiting bacterial attachment to the cell surface, MUC1 may modulate inflammatory responses possibly limiting unnecessary responses when bacteria fail to stably bind to the cell surface. Further work is required to fully define the influence of MUC1 on epithelial cell responses to the presence of *H. pylori*.

Human population studies have found associations between *MUC1* allele polymorphisms and susceptibility to the development of gastric adenocarcinoma and *H. pylori*-associated gastritis [18],[19]. VNTR length polymorphisms have been used to characterize these alleles and short VNTR alleles which encode MUC1 proteins with shorter extracellular glycosylated domains are associated with disease. Our data are consistent with the interpretation that shorter forms of MUC1 are less efficient at sterically inhibiting attachment or acting as releasable decoys, thereby allowing increased bacterial binding to the epithelial surface and exacerbation of pathology. Experiments where cells were transfected with MUC1 expressing differing VNTR lengths suggest that longer alleles are more efficient at binding *H. pylori* [45]. However, it is possible that these VNTR length polymorphisms are surrogate markers for SNP's encoding functional changes in other domains (for example, SNPs affecting cytoplasmic domain signaling or cleavage of the extracellular domain) or promoter polymorphisms affecting the level of MUC1 expression during infection. More comprehensive genetic epidemiological studies are warranted to further define the nature of the *MUC1* risk alleles.

Our experiments show that MUC1 inhibits the *H. pylori* binding to epithelial cells that occurs via both the BabA and SabA adhesins and non-adhesin mediated binding. When the pathogen does not bind to MUC1, the mucin sterically inhibits adhesion to other potential cell surface ligands. When the pathogen does bind to MUC1, the extracellular domain of the mucin is released from the epithelial surface, thereby acting as a releasable decoy and preventing prolonged adherence. Demonstration of the mechanism by which MUC1 limits gastric *H. pylori* infection is a model paradigm for elucidation of the function of the family of cell surface mucins which decorate the apical membrane surface of all mucosal epithelial cells, and for exploration of their contribution to preventing infectious and inflammatory disease.

## Materials and Methods

### Ethics statement

The collection of gastric juice was obtained after written informed consent from patients undergoing upper gastrointestinal endoscopy (approved by the Mater Health Services' Human Research Ethics Committee, Approval No. 396A). All procedures involving animals were reviewed and approved by Institutional animal care and use committees (University of Melbourne; AEEC No. 03219)

### Immunohistochemistry

Dewaxed and rehydrated formalin-fixed sections (4 µm) were treated with 10 mM citric acid, pH 6 at 100°C for 20 min and then with 3% (v/v) hydrogen peroxide for 30 min at room temperature. The sections were washed 3 times between all subsequent steps in 0.15 M NaCl, 0.1 M Tris/HCl buffer (pH 7.4) containing 0.05% Tween-20. Non-specific binding was blocked by protein block (Dako) for 30 min. The sections were incubated with an anti-MUC1 antibody (CT2, gift from Prof. S. Gendler, Scottsdale, USA) diluted 1∶50 in Antibody Diluent (Dako) for 1 h, then incubated with Broad Spectrum Poly HRP Conjugate (Zymed Laboratories Inc, San Fransisco, USA) for 10 min and with diaminobenzidine for 10 min. The sections were counterstained with Harris's haematoxylin.

### MUC1 antibodies

Three antibodies against MUC1 were used in this study: The CT2 antibody is against the cytoplasmic tail of MUC1, whereas the BC2 and BC3 antibodies are against the extracellular domain of MUC1, and react with a VNTR repeat epitope that is exposed even in fully glycosylated MUC1 [46]. These antibodies all react with mature glycosylated MUC1 on the apical surface of human gastric epithelium [46].

### Isolation of MUC1 and assessment of MUC1 glycosylation

Gastric juice was obtained after informed consent from patients undergoing upper gastrointestinal endoscopy. The gastric juice was adjusted to pH 7 with Tris and mixed with equal volume of 150 mmol/L NaCl, 1% NP40, 0.5% deoxycholic acid, 0.1% SDS, 50 mmol/L Tris, pH 7.5 containing Complete protease inhibitor (Roche Diagnostics GmbH, Mannheim, Germany) (RIPA buffer) and incubated for 5 min under agitation at 4°C, centrifuged at 10,000 g for 10 min at 4°C and the supernatant used for the binding assays. Cells from the gastric cancer cell line MKN7 (Riken, Japan) were lysed in RIPA buffer for 10 min under agitation at 4°C, lysates centrifuged at 10,000 g for 10 min at 4°C and the high molecular weight fraction purified from the supernatant using a Microcon centrifugal filter device (cut off 100 kDa). Microtitre plates (Polysorb, Nunc, Denmark) were coated overnight at 4°C with either the BC2 antibody or the isotype control antibody 401.21 against α-gliadin (100 µL/well, 4 µg/mL in phosphate-buffered saline, PBS). The plates were then washed 3 times in PBS with 0.05% Tween-20 and unbound sites blocked with 1% BSA in PBS for 1 h at room temperature. The wells were then incubated with cell lysate or gastric juice extract diluted 1∶5 in Blocking reagent for ELISA (Boehringer Mannheim, Germany) containing 0.5% BSA and 0.05% Tween-20 (dilution buffer) for 2 h with orbital shaking, washed as above, incubated with primary antibody: anti-MUC1 (clone BC3), anti-Le^b^ (clone LE2, Biotest, Dreieich, Germany), anti-sialyl-Le^a^ (clone CA19-9, NeoMarkers, Freemony, CA, USA) or anti-sialyl-Le^x^ (AM3, gift from Dr C. Hanski, University Medical Center Charite, Berlin, Germany) diluted to 1 µg/mL, 1∶200, 1∶1000 and 1∶20, respectively. The plates were washed and incubated with HRP-conjugated anti-mouse IgM (Jackson ImmunoResearch Laboratories, Inc, USA). HRP activity was determined using 2,2′-Azinobis(3-ethylbenzothiazoline)-6-sulphonic acid as a substrate (0.550 g/L in citrate phosphate buffer pH 4.3) by measuring absorbance at 405 nm. Assays were performed in triplicate.

### *H. pylori* adherence to glycoconjugates

*H. pylori* were harvested and washed twice by centrifugation at 2,500 g in PBS containing 0.05% Tween-20. 100 µl *H. pylori* (OD600 nm = 0.90) was incubated with 500 ng FITC labelled Le^b^ or sialyl-Le^x^ HSA-conjugates for 30 min in PBS containing 0.5% human albumin and 0.05% Tween-20. Fluorescence was measured after washing the bacteria twice. Assays were performed in triplicate.

### MUC1 bacterial binding assays

Binding of *H. pylori* to MUC1 was determined by sandwich ELISA using biotinylated bacteria. Polysorb Microtitre plates (Nunc, Denmark) were coated overnight at 4°C with either the BC2 antibody against the extracellular domain of human MUC1, or the isotype control antibody 401.21 (100 µL/well, 4 µg/mL in PBS). The plates were then washed 3 times in PBS with 0.05% Tween-20 and unbound sites blocked with 1% BSA in PBS for 1 h at room temperature. The wells were then incubated with cell lysate or gastric juice extract diluted 1∶5 in Blocking reagent for ELISA (Boehringer Mannheim, Germany) containing 0.5% BSA and 0.05% Tween-20 (dilution buffer) for 2 h with orbital shaking, washed as above, then incubated with a suspension of biotinylated SS1, J99wt or J99ΔBabA/SabA (OD_600_ = 0.15 diluted 1∶10 in dilution buffer) for 2 h at 37°C with orbital shaking. The plates were washed and incubated with streptavidin-HRP (diluted 1∶1000 in dilution buffer) for 1 h at room temperature and HRP activity determined as above. To get a sufficient amount of MUC1 to analyze binding with a range of *H. pylori* strains, archived purified mucins from a previously published study on tissue from patients with no history of peptic ulcer disease undergoing elective surgery for morbid obesity was used [11]. MUC1 was isolated from whole gastric wall using isopycnic density gradient centrifugation followed by gel chromatography of the mucin containing fractions to separate MUC1 from the oligomeric mucins as previously described [9], and analyzed by ELISA [9].

### Cell culture

The gastric epithelial cell line MKN7 (Riken Cell Bank, Japan) was cultured in RPMI containing 10% FCS, 2 mM L-glutamine, 100 units/mL penicillin G sodium and 100 ug/mL streptomycin. Mucin expression and glycosylation in this cell line has been described previously [22]. For co-culture with *H. pylori* the medium was changed to antibiotic free medium 20 h prior to infection. The co-culture experiments were performed on confluent MKN7 cells transferred to microaerobic conditions (5% O_2_, 15% CO_2_, 80% N_2_) at the start of the co-culture. MKN7 cell viability is not compromised during these conditions [22], and the microaerobic conditions are similar to the actual pO_2_ and pCO_2_ in tissues of the human body [47].

To assess integrity of monolayer cultures MKN7 cells were cultured on snapwell tissue culture inserts, which were then were mounted in vertical Ussing chambers (exposed area 1.13 cm^2^). The basolateral side of the membrane was immersed in 115.8 mM NaCl, 1.3 mM CaCl_2_, 3.6 mM KCl, 1.4 mM KH_2_PO_4_, 23.1 mM NaHCO_3_, 1.2 mM MgSO_4_ (KREB) solution containing 5.7 mM Na-pyruvate, 5.1 mM Na-L-glutamate 10 mM and D-glucose, whereas the apical compartment was immersed in KREB's solution containing 5.7 mM Na-pyruvate, 5.13 mM Na-L-glutamate and 10 mM D-mannitol. The solutions were gassed with 95% O_2_ and 5% CO_2_ at a temperature of 37°C and pH 7.4 throughout the whole experiment. Epithelial resistance (Rp) was measured using square-pulse analysis. 5 V, 3 ms pulses were generated by a square pulse generator (Medimet, Gothenburg, Sweden) via a current limiting resistor (36 kΩ) connected to a platinum electrode and applied across the sample.

### *H. pylori* culture

*H. pylori* were grown on Brucella agar supplemented with 10% bovine blood, 2% Vitox (Oxoid), 10 µg/mL vancomycin (Sigma), 5 µg/mL trimethoprim (Sigma) and 4 µg/mL amphoteracin B (Sigma) for 4 days in 5% O_2_ and 15% CO_2_ at 37°C. *H. pylori* strains J99 wild type (wt) bind Le^b^ and sialyl-Le^x^, CCUG17875/Leb and P466 bind Le^b^ but not sialyl-Le^x^, the 17875BabA1::kanbabA2::cam-mutant (75Δ*babA1A2*) and CCUG17874, bind sialyl-Le^x^ but not Le^b^, whereas the remaining strains; the isogenic J99 adhesion mutant lacking the BabA and SabA adhesins (J99*babA*::cam*sabA*::kan [13], referred to as J99ΔBabAΔSabA), 26695, P1 and P1-140 do not bind sialyl-Le^x^ or Le^b^ [11] (provided by Prof. Thomas Boren, Umeå University, Sweden). The J99 CagA deletion mutant (J99ΔCagA) was made by natural transformation with a plasmid containing the cloned CagA from strain 26695 that was knocked out by insertion of the Cat_GC_ cassette into the singular *Bg*/II site in the middle of the *cagA* gene (provided by Prof Steffen Backert, Institut fur Medizinische Microbioligie, Magdeburg, Germany). Clones were selected after culture on 6 µg chloroamphenicol/mL agar. Disruption of CagA was verified by PCR using the 5′-AAAGGATTGTCCCTACAAGAAGC-3′ and 5′-GTAAGCGATTGCTCTTGCATC sequences. The concentration of *H. pylori* was estimated by measuring OD600, and then the amount of CFU in the inoculum was determined by counting colonies from serial dilutions cultured for 5 days. The Multiplicity Of Infection (MOI, pathogen∶host cell) was determined based on the CFU of the *H. pylori* and a density of 1.5×10^5^ MKN7 cells/cm^2^ cells at the day of confluency, which is the day we infected the cells.

### Biotinylation of *H. pylori*

The bacteria were washed twice in 0.2 mol/L carbonate buffer pH 8.3 (2×10^9^ bacteria/mL), 125 µg/mL biotin-XX-NHS was added and the mixture rotated in the dark for 15 min at room temperature. To verify that the BabA adhesin remained functional, adherence to the Le^b^ conjugate was measured (Figure 2).

### Assessment of bead binding to gastric epithelial cells

10^5^ fluorescent beads (Fluospheres NeutrAvidin labelled 1 µm microspheres, Molecular probes) coated with an antibody against the extracellular domain of MUC1 (BC2 antibody) or isotype control (401.21) were added to 96 well plates with confluent MKN7 epithelial cells (N = 9). After incubation for 4.5 h, 24 h and 44 h, cultures were washed 3 times with ice cold PBS and fluorescence was quantified in a FLA5100 (Fujifilm).

### SDS-PAGE and Western blotting

RIPA lysates were subjected to SDS-PAGE in a 4–12% gradient gel. Western blots were cut in the middle (∼70 kDa) and the upper half was stained with an antibody against the MUC1 extracellular domain (BC2) and the lower half was probed for β-actin and detected with fluorescent probes on the Licor Odyssey instrument.

### RNA preparation and real-time PCR

Total RNA was prepared using the RNeasy Mini Kit (Qiagen, Valencia, CA, USA). The quantity and quality of the RNA was determined by spectrophotometry (ND-1000; NanoDrop Technologies Inc., Wilmington, DE). Total RNA (1 µg) from each sample was used for first strand cDNA synthesis using SuperScript™ III reverse transcriptase (Invitrogen) following the manufacturer's instructions. Real-time PCR was monitored by SYBR® Green I fluorescence (Invitrogen) using Platinum ® Taq DNA-Polymerase (Invitrogen) with 3 mM MgCl_2_, 0.2 µM primers, 200 µM dNTPs, and 0.5 U polymerase per reaction (25 µl) under primer-specific conditions. The following experimental protocol for PCR reaction (40 cycles) was performed on a Rotor-Gene 3000 cycler (Corbett Research, Sydney, Australia): denaturation for 15 min at 95°C, followed by 40 amplification cycles at 94°C (20 s), annealing under primer-specific conditions (30 s), and extension for 45 s at 72°C. Primers with the following sequences were chosen: GAPDH: forward: 5′- CCTGTACGCCAACACAGTGC -3′, reverse: 5′- ATACTCCTGCTTGCTGATCC -3′, annealing temperature 60°C. MUC1: forward: 5′- CCCCTATGAGAAGGTTTCTGC-3′, reverse: 5′- ACCTGAGTGGAGTGGAATGG -3′, annealing temperature 60°C. ADAM-17: forward: 5′-ACCTGAAGAGCTTGTTCATCGAG -3′, reverse: 5′-CCATGAAGTGTTCCGATAGATGTC-3′, annealing temperature 60°C. MMP-14: forward: 5′-CCATCATGGCACCCTTTTACC-3′ reverse: 5′-TTATCAGGAACAGAAGGCCGG-3′; annealing temperature 60°C. All Primers were obtained from GeneWorks (Hindmarsh, SA, Australia). To confirm the specificity of the amplified DNA, a melting curve was determined at the end of each run. The reaction efficiency was determined with a dilution series of cDNA containing the PCR products. Genes were normalized to the unregulated housekeeping gene GAPDH and the results were expressed as ratio of target gene and GAPDH expression (arbitrary units). Control experiments were also performed to ensure that GAPDH expression was not differentially regulated under the experimental conditions employed.

### ADAM 17 and MMP-14 gene silencing in MKN7 cells with siRNA

siRNA specific for ADAM17 and MMP-14 as well as scrambled siRNA were chemically synthesized (Dharmacon Research, Lafayette, USA) as a mixture of four siRNAs targeting different regions of the same gene to enhance the silencing performance (21 mers, SMARTpool). MKN 7 cells with a confluence of 70–80% were transfected with either 250 nM of ADAM17 siRNA or MMP 14 siRNA individually or combined both together using Lipofectamine 2000 Reagent (Invitrogen) according to the manufacturer's instructions. 250 nM or 500 nM of scrambled control siRNA were used as negative control. After 48 h of transfection, the MKN7 cells were co-cultured with *H pylori* J99 wild type for a further 8 and 24 h, and the uninfected MKN 7 cells were also cultured for a further 8 and 24 h as a negative control. The MKN7 cells were harvested after 56 and 72 h transfection, the level of knockdown of the ADAM 17 or MMP-14 was detected by quantitative RT-PCR, and the influence of the treatment on MUC1 shedding was monitored by ELISA.

### MUC1 knockdown by siRNA transfection

100 nM 2′-hydroxyl DsiRNA against the target sequence NNGUUCAGUGCCCAGCUCUAC (1∶1) and NNGCACCGACUACUACCAAGA (1∶3) or siCONTROL non-targeted siRNA#2 (Dharmacon) were transfected into MKN7 cells using Lipofectamine 2000. Four days after transfection the level of knockdown was measured using the median fluorescence intensity determined by flow cytometry (below). The 1∶3 siRNA generally gave higher knockdown than the 1∶1 siRNA.

### Flow cytometry

Cell viability was analysed by collecting non-adherent cells together with attached cells harvested with trypsin and counting cell suspensions by flow cytometry on cells stained with 1 µg/mL 7-aminoactinomycin D (7AAD) and Annexin-V-PE Apoptosis Detection Kit I (BD Pharmingen) according to the manufacturers instructions. For MUC1 detection, cells harvested with trypsin and stained with 1 µg/mL 7AAD were either stained without fixation (staining of cell surface structures) or fixed in 1% paraformaldehyde for 5 min on ice and then permeabilized with 0.5% saponin (intracellular and extracellular staining). The cells were then incubated with the anti-MUC1 antibody BC2 or isotype control 401.21 at 3 µg/mL in 1% BSA in PBS for 60 min at 4°C and then with anti-mouse antibody conjugated to Alexa fluor 488 (Invitrogen). For detection of intracellular antigens, washes and antibody incubations were performed in the presence of 0.5% saponin. After staining, all cells were fixed with 1% paraformaldehyde. The analysis was gated to exclude 7AAD positive cells and assessment of staining was performed on a LSRII Flow Cytometer (BD Biosciences, San Jose, USA) using the Diva software (BD Biosciences, San Jose, USA). Bacteria were recovered from the culture medium of MKN7 cells by first sedimenting non-adherent mammalian cells (300 g, 5 min) and then sedimenting bacteria (5000 g, 10 min). Bacteria were then stained with BC2 or 401.21 at 10 µg/mL as above and gated using the SSC and FSC pattern of broth cultured *H. pylori*.

### Assessment of MUC1 binding to *H. pylori* by confocal microscopy

Bacteria prepared and stained for flow cytometry as above were smeared onto charged glass slides, stained with DAPI (0.1 µg/ml) for 15 min, washed with PBS, mounted in Prolong Gold (Invitrogen) and examined using a Zeiss LSM510 confocal microscope with multitracking detecting DAPI (excitation 405 nm, detection 420–480 nm) and FITC (excitation 488 nm detection, LP 505 nm) fluorescence separately.

### Assessment of *H. pylori* binding to gastric epithelial cells

*H. pylori* J99wt, the J99ΔCagA or J99ΔBabAΔSabA were co-cultured at a concentration of 8×10^5^ CFU *H. pylori*/well in a 96 well plate. Co-cultures were washed 3 times with ice cold PBS and then fixed with 4% paraformaldehyde for 20 min on ice. After washing, wells were blocked for 1 h with 1% BSA in PBS and then incubated with mouse polyclonal anti-*Helicobacter* antisera [20]. The plates were washed and incubated with HRP-conjugated anti-mouse IgG (Jackson ImmunoResearch Laboratories, Inc, USA). The plates were washed and HRP activity determined using TMB by measuring absorbance at 450 nm.

### Experimental animals and procedures

All procedures involving animals were reviewed and approved by Institutional animal care and use committees (University of Melbourne; AEEC No. 03219). The mouse infection tissue samples were archived material from a previously published study [20]. Female aged matched 129/SvJ wild type and 129/SvJ *Muc1^−/−^* mice were infected intra-gastrically once with 10^7^ *H pylori* suspended in 0.1 mL Brain Heart Infusion (Oxoid).

### TUNEL assay

The assay was performed according to the manufacturer's instructions (Roche), except that the reaction was diluted 1∶4 in 0.1 M sodium cacodylate buffer, pH 7.3 to decrease the background. The total number of TUNEL positive cells per 10 randomly selected fields of view in the entire gastric mucosa was counted at 20× magnification.

### Statistics

For normally distributed data the Students t-test was used to compare groups. For analyses where a normal distribution could not be demonstrated, including where the number of replicates was low, the non-parametric Mann Whitney U test was used to compare groups. The ANOVA test was used when comparing 3 or more groups, and to ascertain that the multiple testing did not add to the chance of finding statistically significant differences, the Tukey's or Bonferroni's post hoc tests were used.

## Supporting Information

Figure S1 MUC1 binding of additional *H. pylori* strains. *H. pylori* adhesion to Le^b^ positive, sLe^x^ negative MUC1 isolated from a healthy gastric specimen. MUC1 was isolated using isopychnic density gradient centrifugation followed by gel chromatograpy of the mucin containing fractions to separate MUC1 from the oligomeric mucins as previously described [9], and analyzed by ELISA [9]. CCUG17875/Leb and P466 bind Le^b^ but not sialyl-Le^x^, the 75Δ*babA1A2* and CCUG17874, bind sialyl-Le^x^ but not Le^b^, whereas the remaining strains 26695, P1 and its isogenic mutant P1-140 does not bind neither sialyl-Le^x^ nor Le^b^ [11]. Data are presented as Mean ± SEM binding to the 4 fractions containing MUC1 after subtraction of the mean value of the control wells (bacteria binding to non-mucin containing fractions from the gel chromatogaphy) for that *H. pylori* strain.
(0.17 MB TIF)

Figure S2 Confocal microscopy images of MUC1 binding to *H. pylori* which were recovered from the culture medium of MKN7 cells after 8 h of co-culture. *H. pylori* J99 wild type (A–C) or J99ΔBabAΔSabA (D, E) were either stained with MUC1 extracellular domain antibody BC2 (A, B, D and E) or an isotope control antibody 401.21 (C), followed by a FITC-conjugated secondary antibody (green), and DNA stained with DAPI (blue). Scale bars are shown.
(1.32 MB PDF)

Figure S3 Photomicrographs of Tunel stained sections. Tunel stained sections from stomach of wild-type (*Muc1* ^+/+^) and *Muc1* ^−/−^ mice infected with *H. pylori*-SS1 for 1 week vs 8 weeks.
(9.84 MB TIF)

## References

[1] Blaser MJ, Atherton JC (2004). Helicobacter pylori persistence: biology and disease.. J Clin Invest.

[2] Targa AC, Cesar AC, Cury PM, Silva AE (2007). Apoptosis in different gastric lesions and gastric cancer: relationship with Helicobacter pylori, overexpression of p53 and aneuploidy.. Genet Mol Res.

[3] Peek RM, Wirth HP, Moss SF, Yang M, Abdalla AM (2000). *Helicobacter pylori* alters gastric epithelial cell cycle events and gastrin secretion in Mongolian gerbils.. Gastroenterology.

[4] Moss SF, Calam J, Agarwal B, Wang S, Holt PR (1996). Induction of gastric epithelial apoptosis by *Helicobacter pylori*.. Gut.

[5] Handa O, Naito Y, Yoshikawa T (2007). CagA protein of *Helicobacter pylori*: a hijacker of gastric epithelial cell signaling.. Biochem Pharmacol.

[6] Cabral MM, Mendes CM, Castro LP, Cartelle CT, Guerra J (2006). Apoptosis in *Helicobacter pylori* gastritis is related to cagA status.. Helicobacter.

[7] Kato K, Hasui K, Wang J, Kawano Y, Aikou T (2008). Homeostatic Mass Control in Gastric Non-Neoplastic Epithelia under Infection of *Helicobacter pylori*: An Immunohistochemical Analysis of Cell Growth, Stem Cells and Programmed Cell Death.. Acta Histochem Cytochem.

[8] Houghton J, Stoicov C, Nomura S, Rogers AB, Carlson J (2004). Gastric cancer originating from bone marrow-derived cells.. Science.

[9] Linden S, Mahdavi J, Hedenbro J, Boren T, Carlstedt I (2004). Effects of pH on *Helicobacter pylori* binding to human gastric mucins: identification of binding to non-MUC5AC mucins.. Biochem J.

[10] Linden SK, Wickstrom C, Lindell G, Gilshenan K, Carlstedt I (2008). Four modes of adhesion are used during *Helicobacter pylori* binding to human mucins in the oral and gastric niches.. Helicobacter.

[11] Lindén S, Nordman H, Hedenbro J, Hurtig M, Borén T (2002). Strain- and blood group-dependent binding of *Helicobacter pylori* to human gastric MUC5AC glycoforms.. Gastroenterology.

[12] Ilver D, Arnqvist A, Ogren J, Frick IM, Kersulyte D (1998). *Helicobacter pylori* adhesin binding fucosylated histo-blood group antigens revealed by retagging.. Science.

[13] Mahdavi J, Sonden B, Hurtig M, Olfat FO, Forsberg L (2002). *Helicobacter pylori* SabA adhesin in persistent infection and chronic inflammation.. Science.

[14] Linden S, Mahdavi J, Semino-Mora C, Olsen C, Carlstedt I (2008). Role of ABO secretor status in mucosal innate immunity and *H. pylori* infection.. PLoS Pathog.

[15] Mahdavi J, Sonden B, Hurtig M, Olfat FO, Forsberg L (2002). *Helicobacter pylori* SabA adhesin in persistent infection and chronic inflammation.. Science.

[16] Ota H, Nakayama J, Momose M, Hayama M, Akamatsu T (1998). *Helicobacter pylori* infection produces reversible glycosylation changes to gastric mucins.. Virchows Arch.

[17] Packer LM, Williams SJ, Callaghan S, Gotley DC, McGuckin MA (2004). Expression of the cell surface mucin gene family in adenocarcinomas.. Int J Oncol.

[18] Carvalho F, Seruca R, David L, Amorim A, Seixas M (1997). Muc1 gene polymorphism and gastric cancer - an epidemiological study.. Glycoconjugate J.

[19] Vinall LE, King M, Novelli M, Green CA, Daniels G (2002). Altered expression and allelic association of the hypervariable membrane mucin MUC1 in *Helicobacter pylori* gastritis.. Gastroenterology.

[20] McGuckin MA, Every AL, Skene CD, Linden SK, Chionh YT (2007). Muc1 mucin limits both *Helicobacter pylori* colonization of the murine gastric mucosa and associated gastritis.. Gastroenterology.

[21] McAuley JL, Linden SK, Png CW, King RM, Pennington HL (2007). MUC1 cell surface mucin is a critical element of the mucosal barrier to infection.. J Clin Invest.

[22] Linden SK, Driessen KM, McGuckin MA (2007). Improved in vitro model systems for gastrointestinal infection by choice of cell line, pH, microaerobic conditions, and optimization of culture conditions.. Helicobacter.

[23] Kwok T, Zabler D, Urman S, Rohde M, Hartig R (2007). *Helicobacter* exploits integrin for type IV secretion and kinase activation.. Nature.

[24] Thathiah A, Blobel CP, Carson DD (2003). Tumor necrosis factor-alpha converting enzyme/ADAM 17 mediates MUC1 shedding.. J Biol Chem.

[25] Thathiah A, Carson DD (2004). MT1-MMP mediates MUC1 shedding independent of TACE/ADAM17.. Biochem J.

[26] Viala J, Chaput C, Boneca IG, Cardona A, Girardin SE (2004). Nod1 responds to peptidoglycan delivered by the *Helicobacter pylori* cag pathogenicity island.. Nat Immunol.

[27] Minohara Y, Boyd DK, Hawkins HK, Ernst PB, Patel J (2007). The effect of the cag pathogenicity island on binding of *Helicobacter pylori* to gastric epithelial cells and the subsequent induction of apoptosis.. Helicobacter.

[28] Yu J, Leung WK, Go MY, Chan MC, To KF (2002). Relationship between *Helicobacter pylori* babA2 status with gastric epithelial cell turnover and premalignant gastric lesions.. Gut.

[29] Yanai A, Maeda S, Hikiba Y, Shibata W, Ohmae T (2007). Clinical relevance of *Helicobacter pylori* sabA genotype in Japanese clinical isolates.. J Gastroenterol Hepatol.

[30] Lindén S, Borén T, Dubois A, Carlstedt I (2004). Rhesus monkey gastric mucins and their *Helicobacter pylori* binding properties.. Biochem J.

[31] El Miedany YM, Baddour M, Ahmed I, Fahmy H (2005). Sjogren's syndrome: concomitant *H. pylori* infection and possible correlation with clinical parameters.. Joint Bone Spine.

[32] Vinall LE, King M, Novelli M, Green CA, Daniels G (2002). Altered expression and allelic association of the hypervariable membrane mucin MUC1 in *Helicobacter pylori* gastritis.. Gastroenterology.

[33] Byrd JC, Yunker CK, Xu QS, Sternberg LR, Bresalier RS (2000). Inhibition of gastric mucin synthesis by Helicobacter pylori.. Gastroenterology.

[34] Cottet S, Corthesy-Theulaz I, Spertini F, Corthesy B (2002). Microaerophilic conditions permit to mimic in vitro events occurring during in vivo *Helicobacter pylori* infection and to identify Rho/Ras-associated proteins in cellular signaling.. J Biol Chem.

[35] Macao B, Johansson DG, Hansson GC, Hard T (2006). Autoproteolysis coupled to protein folding in the SEA domain of the membrane-bound MUC1 mucin.. Nat Struct Mol Biol.

[36] Wreschner DH, McGuckin MA, Williams SJ, Baruch A, Yoeli M (2002). Generation of ligand-receptor alliances by “SEA” module-mediated cleavage of membrane-associated mucin proteins.. Protein Sci.

[37] Imbert Y, Darling DS, Jumblatt MM, Foulks GN, Couzin EG (2006). MUC1 splice variants in human ocular surface tissues: possible differences between dry eye patients and normal controls.. Exp Eye Res.

[38] Wei X, Xu H, Kufe D (2005). Human MUC1 oncoprotein regulates p53-responsive gene transcription in the genotoxic stress response.. Cancer Cell.

[39] Cerella C, Mearelli C, Ammendola S, De Nicola M, D'Alessio M (2006). Molecular determinants involved in the increase of damage-induced apoptosis and delay of secondary necrosis due to inhibition of mono(ADP-ribosyl)ation.. Ann N Y Acad Sci.

[40] Huang L, Chen D, Liu D, Yin L, Kharbanda S (2005). MUC1 oncoprotein blocks glycogen synthase kinase 3beta-mediated phosphorylation and degradation of beta-catenin.. Cancer Res.

[41] Foley PJ, Scheri RP, Smolock CJ, Pippin J, Green DW (2008). Targeted Suppression of beta-Catenin Blocks Intestinal Adenoma Formation in APC Min Mice.. J Gastrointest Surg.

[42] Ahmad R, Raina D, Trivedi V, Ren J, Rajabi H (2007). MUC1 oncoprotein activates the IkappaB kinase beta complex and constitutive NF-kappaB signalling.. Nat Cell Biol.

[43] Lu W, Hisatsune A, Koga T, Kato K, Kuwahara I (2006). Cutting edge: enhanced pulmonary clearance of *Pseudomonas aeruginosa* by Muc1 knockout mice.. J Immunol.

[44] Lillehoj EP, Kim BT, Kim KC (2002). Identification of *Pseudomonas aeruginosa* flagellin as an adhesin for Muc1 mucin.. Am J Physiol Lung Cell Mol Physiol.

[45] Costa NR, Mendes N, Marcos NT, Reis CA, Caffrey T (2008). Relevance of MUC1 mucin variable number of tandem repeats polymorphism in *H pylori* adhesion to gastric epithelial cells.. World J Gastroenterol.

[46] Price MR, Rye PD, Petrakou E, Murray A, Brady K (1998). Summary report on the ISOBM TD-4 Workshop: analysis of 56 monoclonal antibodies against the MUC1 mucin. San Diego, Calif., November 17–23, 1996.. Tumour Biol.

[47] Pruden E, Siggard-Andersen O, Tietz N, Tietz N (1986). Blood gases and pH.. Textbook of Clinical Chemistry.

