# Supplementary figures and images for: MUC1 Limits Helicobacter pylori Infection both by Steric Hindrance and by Acting as a Releasable Decoy
Source: PLoS Pathog. 2009 Oct 9;5(10):e1000617. doi: 10.1371/journal.ppat.1000617 (PMC2752161; doi:10.1371/journal.ppat.1000617)

**MUC1 (Fitc)****DNA (Dapi)****Merge**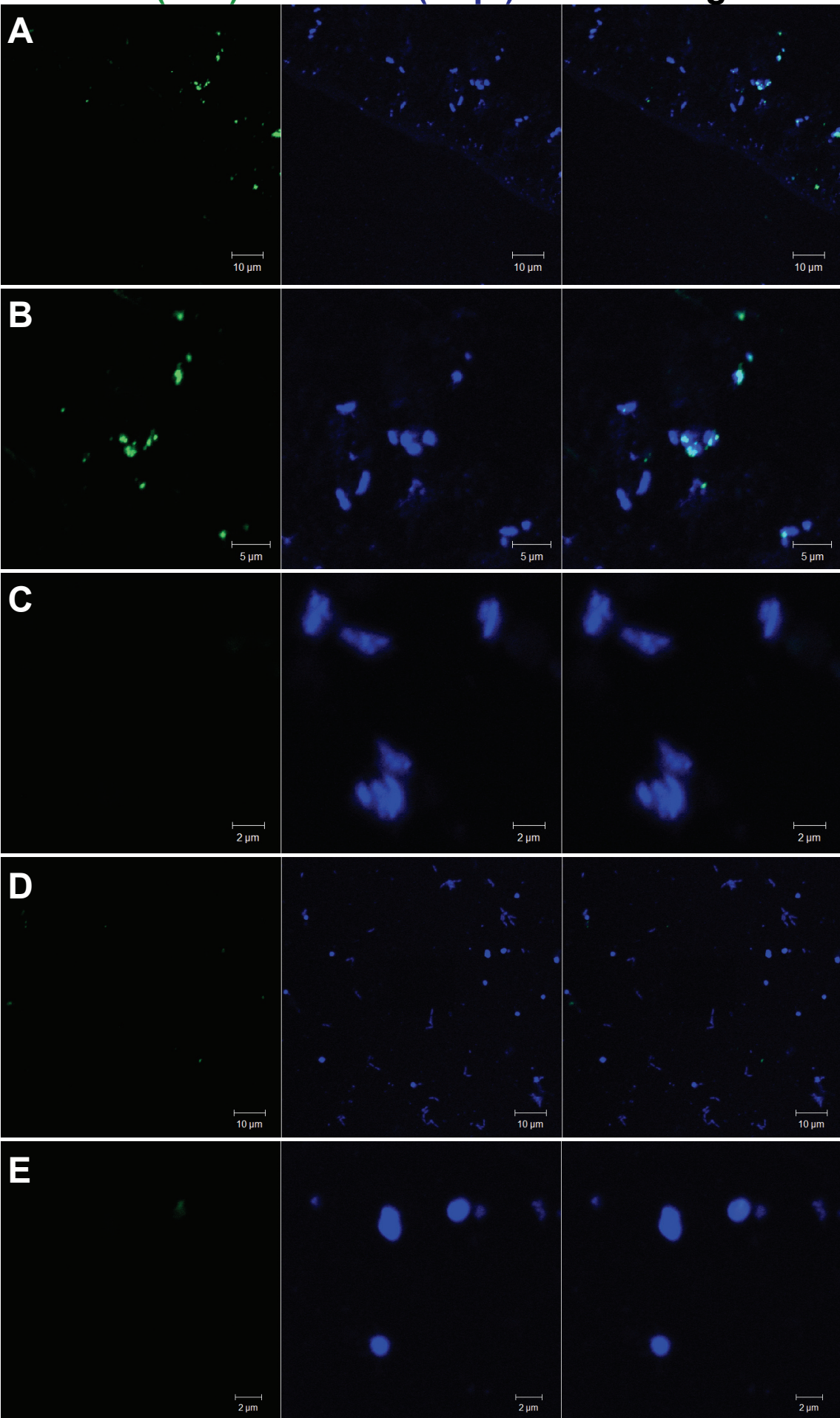

Supplement: Figure S2 — Confocal microscopy images of MUC1 binding to H. pylori which were recovered from the culture medium of MKN7 cells after 8 h of co-culture. H. pylori J99 wild type (A–C) or J99ΔBabAΔSabA (D, E) were either stained with MUC1 extracellular domain antibody BC2 (A, B, D and E) or an isotope control antibody 401.21 (C), followed by a FITC-conjugated secondary antibody (green), and DNA stained with DAPI (blue). Scale bars are shown. (1.32 MB PDF) [file ppat.1000617.s002.pdf]

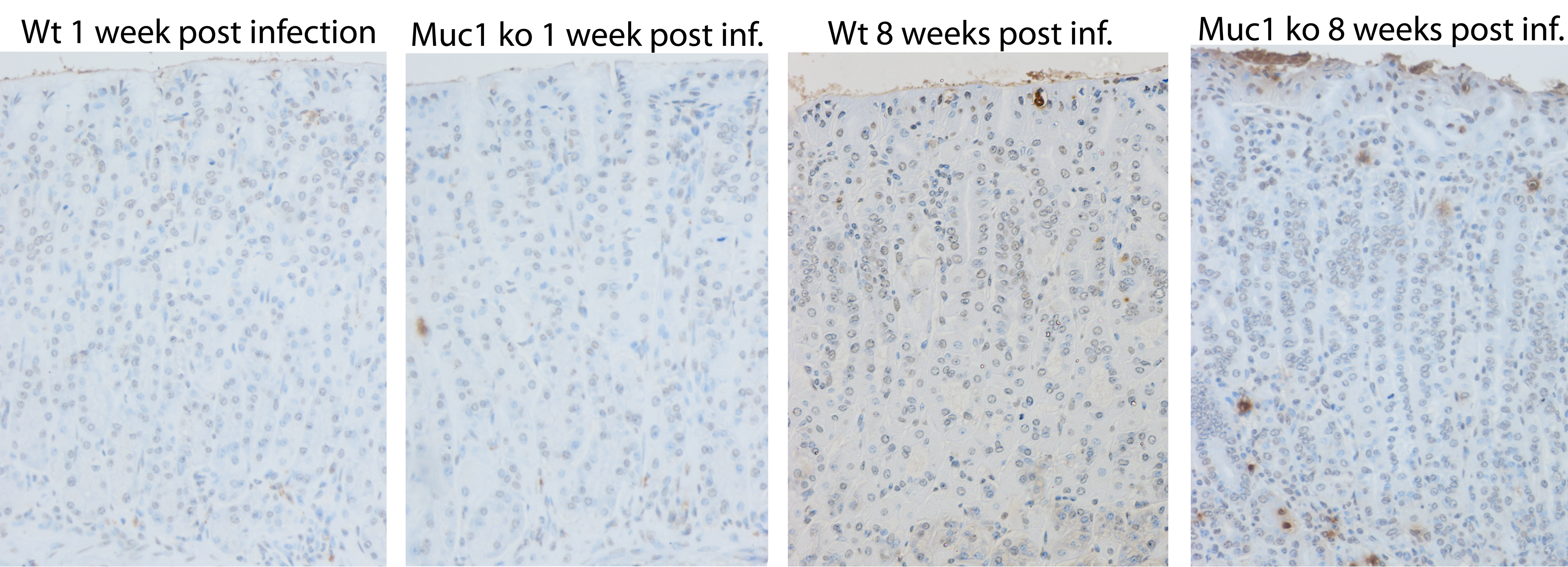

Supplement: Figure S3 — Photomicrographs of Tunel stained sections. Tunel stained sections from stomach of wild-type (Muc1 +/+) and Muc1 −/− mice infected with H. pylori-SS1 for 1 week vs 8 weeks. (9.84 MB TIF) [file ppat.1000617.s003.tif]
